# Encoding Effort Eliminates the Animacy Advantage in Memory When Manipulated with Value-Directed Remembering

**DOI:** 10.3390/bs16010030

**Published:** 2025-12-23

**Authors:** Julia N. Keiner, Nicolasa C. Villalobos, T. D. Kelley, Michael J. Serra

**Affiliations:** 1 Department of Psychological Sciences, College of Arts & Sciences, Texas Tech University, Lubbock, TX 79409, USA; jushull@ttu.edu (J.N.K.); nicovill@ttu.edu (N.C.V.); 2 Jane and Terry Semel Institute for Neuroscience and Human Behavior, University of California Los Angeles, Los Angeles, CA 90095, USA; 3 Department of Medical Education, School of Medicine, Texas Tech University Health Sciences Center, Lubbock, TX 79430, USA

**Keywords:** animacy, animacy advantage, animacy effect, free-recall performance, encoding effort, value-directed remembering, adaptive memory

## Abstract

People are more likely to remember words that refer to living/animate things than nonliving/inanimate things across various memory tasks, yielding an animacy advantage in recall. We tested an encoding-effort explanation for this effect: that people naturally devote more attention or encoding effort to living things over nonliving things during encoding, producing the effect. We used both between-participants (Experiment 1) and within-participants (Experiment 2) manipulations of value (i.e., participants earned a different number of points for each word they recalled) to affect encoding effort at the task and item level, respectively. We predicted that leading participants to devote more encoding effort to the items (in particular, the inanimate words) would reduce or even eliminate the animacy advantage, as has been found in some prior studies that used mental-imagery manipulations. Overall, participants recalled more animate words than inanimate words and recalled more words under higher-effort than under lower-effort conditions. In line with our predictions, these two factors interacted: the animacy effect was eliminated under higher-effort conditions, as these conditions led to an increase in the recall of inanimate words compared to animate words. The results therefore support an encoding-effort explanation for the animacy advantage in free-recall performance.

## 1. Introduction

The term “animacy” refers to the properties that lead us to consider something alive, distinguishing living from nonliving items (Serra et al., 2024). Animate things include animals and people (e.g., butterfly; doctor), while inanimate things include natural and manmade objects (e.g., sand; car). People often demonstrate an “animacy advantage” in memory—recalling more animate than inanimate items—across various tasks. This effect has been observed in recognition (Bonin et al., 2014; VanArsdall et al., 2013; but see Leding, 2020) and some cued-recall tasks (e.g., DeYoung & Serra, 2021; Mah et al., 2023; Popp & Serra, 2016; VanArsdall et al., 2015; but see Kazanas et al., 2020; Popp & Serra, 2016; Serra & DeYoung, 2023b). The present experiments focus on the animacy advantage in free recall (e.g., Bonin et al., 2015; Félix et al., 2019; Gelin et al., 2017, 2019; Leding, 2018, 2019; Li et al., 2016; Mah et al., 2023; Meinhardt et al., 2018, 2020; Nairne et al., 2013, 2017; Popp & Serra, 2016, 2018; Serra, 2021; Serra & DeYoung, 2023a; VanArsdall et al., 2017).

In typical free-recall tasks, participants study a list of words presented one at a time, then attempt to recall them without cues. Participants typically recall more animate words than inanimate words in such tasks (Nairne et al., 2013). This advantage holds across serial position (Serra, 2021), with (Nairne et al., 2013) and without (Popp & Serra, 2016) buffer words or a distractor task, for both pure and mixed lists (Komar et al., 2023; Popp & Serra, 2016), under incidental and intentional learning conditions (Félix et al., 2019), and with both computer-paced and self-paced study (Serra & DeYoung, 2023a). Despite its robustness, no single mechanism has been identified as the primary cause.

### 1.1. Potential Mechanisms

Early accounts of the animacy advantage emphasized adaptive explanations, proposing that evolutionary pressures shaped memory processes to prioritize information about living things (cf. Nairne et al., 2013, 2017; New et al., 2007; Serra et al., 2024). Such accounts suggest potential proximal mechanisms that could help to explain the effect. For example, animate things capture our attention more than inanimate ones (e.g., Altman et al., 2016; Bugaiska et al., 2019; Calvillo & Jackson, 2014; Guerrero & Calvillo, 2016), leading many to suggest that attention contributes to the animacy advantage in memory (cf. Bonin et al., 2015; Bugaiska et al., 2019; Félix et al., 2019; Gelin et al., 2017; Leding, 2019, 2020; Nairne et al., 2013; Popp & Serra, 2016; Rawlinson & Kelley, 2021). To test attention-based explanations of the effect, some studies imposed a cognitive load during encoding. For example, Bonin et al. (2015) required participants to maintain irrelevant digit strings during encoding, while Rawlinson and Kelley (2021) used a monitoring task. Both studies predicted that a cognitive load would amplify the animacy advantage by diverting resources away from the processing of inanimate words (lowering their recall) while animate words would still receive prioritized processing (not affecting their recall). The results, however, did not support this prediction. Both manipulations reduced overall recall but left the animacy advantage intact. Leding (2019) also imposed a monitoring task, finding a reduced—but not fully eliminated—animacy advantage. Crucially, no study has shown that cognitive load increases the animacy advantage as predicted by these accounts.

Researchers have eliminated other explanations for the animacy advantage. Arousal enhances memory (Osugi & Ohira, 2017; Sharot & Phelps, 2004), and animate words tend to be more arousing than inanimate words (Leding, 2019; Popp & Serra, 2018), but emotional arousal (Meinhardt et al., 2018), mental arousal (Popp & Serra, 2018), and threat (Leding, 2019, 2020) are not major contributors to the effect. It also does not result from categorical encoding or retrieval strategies (Gelin et al., 2017; Serra, 2021; VanArsdall et al., 2017) or from participants’ beliefs about animacy (DeYoung & Serra, 2021). Evidence is mixed as to whether greater imagery for animate items contributes to the effect (Blunt & VanArsdall, 2021; Bonin et al., 2015; Gelin et al., 2019; Serra et al., 2025).

Most relevant to the present study is a broad class of effortful-encoding explanations for the animacy advantage that researchers have also described as “richness of encoding” (cf. Bonin et al., 2022; Meinhardt et al., 2020) or “controlled processing” (cf. Rawlinson & Kelley, 2021). Regardless of the label, the basic idea of such accounts is that animate items might invoke more (quantitative) or deeper (qualitative) processing than inanimate items during encoding, leading to greater recall for the animate items. For example, Meinhardt et al. (2020) found that animate words caused more related thoughts during encoding than did inanimate words; the number of thoughts partially mediated the animacy advantage (but see Bonin et al., 2022). Similarly, Rawlinson and Kelley (2021) showed that animate concepts tend to have more semantic features than do inanimate concepts; the number of semantic features also partially mediated the effect of animacy on free-recall performance. Although these findings support at least two versions of an effortful-encoding account for the animacy advantage in free-recall, such mechanisms need not operate alone (e.g., animate items could concurrently elicit more related thoughts and have more semantic features than inanimate items, both of which can enhance memory).

Several studies have tested a specific prediction of effortful-encoding accounts: that the animacy advantage should be smaller under conditions involving high cognitive load because it will make participants less able to process the animate words more deeply than the inanimate words (cf. Rawlinson & Kelley, 2021). Although Leding (2019) found that a cognitive load did reduce the size of the animacy advantage, both Bonin et al. (2015) and Rawlinson and Kelley (2021) found that the advantage maintained under cognitive load. If, however, the effortful encoding in question requires minimal cognitive resources (i.e., if it is “automatic”), then imposing cognitive load might not affect this tendency.

**Figure 1:**
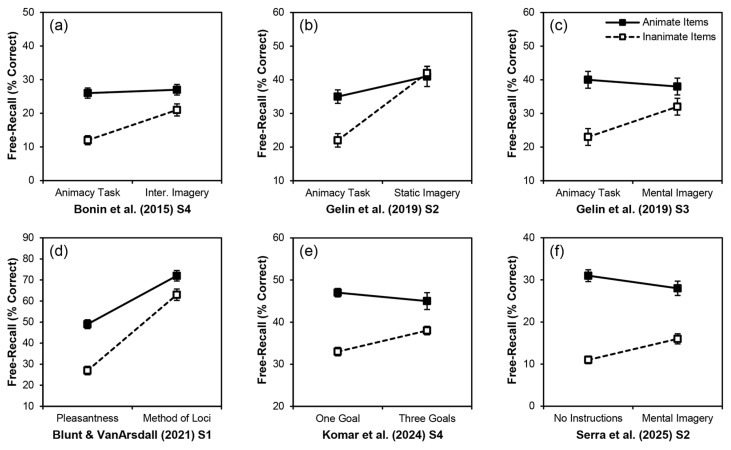
Mean free-recall performance (% correct) for animate and inanimate items, split by encoding condition, from some prior studies that found an interaction of animacy with a presumed manipulation of encoding effort. Solid black lines and black boxes indicate animate items; dashed black lines and white boxes indicate inanimate items. Error bars represent one standard error of the mean. (**a**) Bonin et al. (2015) Study 4; (**b**) Gelin et al. (2019) Study 2; (**c**) Gelin et al. (2019) Study 3; (**d**) Blunt and VanArsdall (2021) Study 1; (**e**) Komar et al. (2024) Study 4; (**f**) Serra et al. (2025) Study 2.

Crossing animacy with another factor that leads to the differential processing of items such as an instructional manipulation (e.g., Murphy & Castel, 2021; Sahakyan & Kelley, 2002) might be a better alternative. Rather than trying to decrease the recall of animate items, studies using an effortful-processing manipulation try to increase the recall of inanimate items. Figure 1 summarizes several studies that have taken such an approach—either intentionally or inadvertently while testing other related hypotheses—and found a significant interaction of animacy with a factor that likely affected encoding effort. In each panel, we present the condition that presumably involved more effort (and showed a reduced animacy advantage) on the right. Five involved mental-imagery manipulations.

In Bonin et al. (2015, Study 4; Figure 1a), the participants either rated the animacy of animate and inanimate words (less effort) or engaged in interactive imagery (greater effort) for animate and inanimate words. Both groups then completed a surprise free-recall test. The interaction of animacy and mental imagery was significant, as the recall of animate words was comparable across conditions, but the recall of inanimate words was increased by mental imagery. In two similar studies, Gelin et al. (2019, Studies 2 & 3; Figure 1b,c) also found significant interactions between animacy and visual imagery, with imagery increasing the recall of inanimate words. Blunt and VanArsdall (2021, Study 1; Figure 1d) either had participants rate the pleasantness of animate and inanimate words or engage in the method of loci before free recall. The method of loci greatly increased the recall of both animate and inanimate items, but nevertheless there was a significant interaction of animacy with instructions such that the recall of inanimate words was closer to that of animate words when participants used the method of loci. Komar et al. (2024, Study 4; Figure 1e) adapted a survival-processing scenario: participants in their three-goals condition had to process each word for its survival relevance in terms of obtaining food, obtaining water, and protecting themselves from predators, whereas those in the one-goal condition only had to process each word for its relevance for obtaining water. They then completed a surprise free-recall test. They obtained an interaction such that the animacy advantage was reduced for the three-goals condition. Much as in Figure 1a–c, Serra et al. (2025, Study 2; Figure 1f) also obtained an interaction such that the animacy advantage was reduced by mental imagery instructions.

Despite such results, which support encoding-effort explanations for the animacy advantage, other studies have obtained contradictory data. Kamp et al. (2025), for example, examined neurological bases of the effect using electroencephalography (EEG). Although they obtained the animacy advantage in both recall and recognition, event-related potentials (ERP) suggested that it does not stem from greater encoding effort for animate versus inanimate items. Instead, the results suggested that memory for animate words is advantaged due to greater semantic access to animate words during encoding and activation of a more complex memory trace for animate words during retrieval.

**Figure 2:**
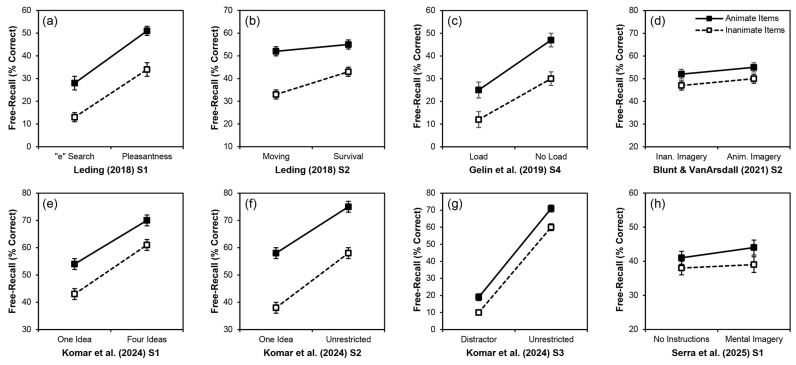
Mean free-recall performance (% correct) for animate and inanimate items, split by encoding condition, from some prior studies that did not find an interaction of animacy with a presumed manipulation of encoding effort. Solid black lines and black boxes indicate animate items; dashed black lines and white boxes indicate inanimate items. Error bars represent one standard error of the mean. (**a**) Leding (2018) Study 1; (**b**) Leding (2018) Study 2; (**c**) Gelin et al. (2019) Study 4; (**d**) Blunt and VanArsdall (2021) Study 2; (**e**) Komar et al. (2024) Study 1; (**f**) Komar et al. (2024) Study 2; (**g**) Komar et al. (2024) Study 3; (**h**) Serra et al. (2025) Study 1.

Other studies, more like those in Figure 1 (including studies from the same papers), have used manipulations that presumably involved more effortful processing, yet failed to obtain an interaction of animacy and presumed encoding effort (Figure 2). Leding (2018) crossed animacy with some depth-of-processing manipulations: indicating if each word contained the letter “e” versus pleasantness ratings in Study 1 (Figure 2a) and rating moving relevance versus survival relevance in Study 2 (Figure 2b). Both effortful encoding conditions led to greater recall performance on a surprise free-recall test than did the respective less effortful conditions, but interactions of animacy and effort did not occur in either case. Much like Bonin et al. (2015) and Rawlinson and Kelley (2021), Gelin et al. (2019, Study 4; Figure 2c) involved a cognitive load, but it failed to alter the animacy advantage. Blunt and VanArsdall’s (2021) Study 2 (Figure 2d) involved two different mental-imagery conditions: imagining the target doing something that suggested it was animate (e.g., crawling towards me) or inanimate (e.g., being made of silver). Although the animate imagery condition produced significantly higher recall than the inanimate imagery condition, animacy and imagery type did not interact: the recall of animate words was consistently higher than for inanimate words regardless of the type of imagery participants engaged in. Komar et al. (2024) conducted four studies that were direct tests of encoding-effort explanations for the animacy advantage. Although the critical interaction of animacy with effort occurred in their fourth study (see Figure 1e), it did not occur in their first three studies (see Figure 2e–g). Those studies involved having participants generate one versus four ideas for each word (Study 1), generate one versus unlimited ideas (Study 2), and complete a distractor task versus generate unlimited ideas (Study 3). Finally, although Serra et al. (2025, Study 2; Figure 1f) obtained a significant interaction of animacy with mental imagery, that study involved implicit encoding. Serra et al. (2025, Study 1; Figure 2h) used explicit instructions and did not obtain an interaction.

Taken together, evidence from various published studies (including and in addition to those summarized in Figure 1 and Figure 2) both support and do not support effortful encoding explanations for the animacy advantage in free-recall performance. We conducted the present experiments as further tests of this explanation.

### 1.2. The Present Experiments

We tested the effortful-encoding explanation for the animacy advantage in free-recall performance. It is difficult to directly manipulate encoding effort, especially under incidental learning conditions as in most previous tests of this explanation. Instead, note that the studies in Figure 1 and Figure 2—regardless of whether their intention was testing encoding-effort explanations for the effect—used manipulations that likely led participants to exert greater effort under one condition than another, often in an indirect way.

To manipulate (presumed) encoding effort in these experiments, we adapted methodology from research on value-directed remembering, which is the capacity to prioritize and recall information that carries greater value or reward when one likely cannot remember all of the items (e.g., Ariel et al., 2009; Hennessee et al., 2019; Knowlton & Castel, 2022; Murphy, 2023; Murphy & Castel, 2022). Experiments typically examine value-directed remembering by randomly assigning different point values or monetary rewards to different items, earned by successfully recalling or recognizing them (e.g., Castel et al., 2002; Elliott & Brewer, 2019; Hennessee et al., 2019; Murphy et al., 2024). When participants cannot easily remember all the items, they prioritize high-value over low-value items to maximize their rewards (e.g., Knowlton & Castel, 2022; Madan, 2017; Schultz, 2015).

Although some studies conclude that retrieval contributes to such selective-memory effects (Murphy & Castel, 2022; Murphy et al., 2024; Stefanidi et al., 2018), evidence more strongly indicates that differences in strategic allocation of attention or effort to higher- and lower-value items during encoding plays a larger and more critical role in producing these effects (Ariel et al., 2009, 2015; Castel, 2008; McGillivray & Castel, 2011; Murphy et al., 2024; Schwartz et al., 2020, 2023). Value-directed remembering is therefore an ideal methodology for testing the present hypothesis. Unlike instruction-based manipulations, which might often need to be enacted using between-participants designs, value-directed remembering can more easily be used in a within-participants manner, as values can be randomly assigned to specific items. We can therefore use value-directed remembering to alter one group’s attention and effort compared to another group’s and we can even alter the attention and effort that participants allocate to specific items. This is the approach that we used in the present Experiments 1 and 2, respectively. We ran these two experiments in tandem, randomly assigning each participant to either of the two groups in Experiment 1 or to the single group in Experiment 2 at the onset of the task.

## 2. Experiment 1

Given that most of the studies we have reviewed involved between-participants manipulations of factors that likely led to greater processing of the items under one condition than the other, we used a between-participants manipulation of encoding effort in the first experiment. More specifically, for the Even Points Group, all items were worth six points if recalled correctly and the instructions noted that participants should attempt to maximize their score by memorizing as many items as possible. In contrast, there was no mention of points for the No Points Group. This manipulation might lead to higher recall for the Even Points Group than for the No Points Group, but this outcome was not critical for the present hypothesis. More important, we predicted that animacy and group (i.e., effort) would interact such that the animacy advantage would be reduced or even eliminated for the Even Points Group, much as in the studies in Figure 1.

### 2.1. Materials and Methods

All materials, data, code, and protocols from the experiments are available on OSF at https://osf.io/zthx2/overview?view_only=948f998168fd4604a48a8f90f4f549b6 (accessed on 29 October 2025).

The present experiments were approved by the IRB at Texas Tech University prior to their occurrence via a blanket protocol for memory tasks (07/19/12, IRB# 503507). The researchers obtained informed consent from all participants at the start of the procedure.

#### 2.1.1. Design

This experiment utilized a 2 (effort level: lower effort vs. higher effort) × 2 (animacy: animate items vs. inanimate items) × 3 (trial: 1 v. 2 vs. 3) mixed-effects design. Effort level was a between-participants variable (i.e., No Points v. Even Points), whereas animacy and trial were both within-participants variables.

#### 2.1.2. Participants

We used G*Power (Faul et al., 2007) to conduct an a priori power analysis to determine the target sample size for Experiment 1. We used parameters from Serra et al. (2025, Study 2) as the basis for these calculations, as that experiment obtained a significant interaction of animacy with presumed effort (*η_p_*^2^ = 0.093, or *f*(*U*) = 0.320). Under the “ANOVA: repeated measures, within-between interaction” menu, we indicated two groups and two measurements. G*Power indicated a sample size of 132 participants to achieve statistical power of 0.95 for the interaction.

We recruited 120 undergraduate students from Texas Tech University who participated for partial or extra credit in a course. Although this study involved “earning points” for correctly recalling items, these points had no relevance for receiving credit; all participants received the same credit for simply participating, not based on their level of performance. Mean age was 19.23 years (*SD* = 1.34). Of the participants, 91 (75.8%) identified as female, 26 (21.7%) as male, 1 (0.8%) as nonbinary, and 2 (1.7%) did not respond. For race/ethnicity, 65 (54.2%) identified as white or Caucasian; 26 (21.7%) as Hispanic or Latino; 10 (8.3%) as black or African American; 7 (5.8%) as Asian or Asian American; 5 (4.2%) as multiracial; 4 (3.3%) as “other”; and 3 (2.5%) did not respond.

#### 2.1.3. Materials

Study materials were the 40 animate and 40 inanimate words from Popp and Serra (2018). Those authors matched the inanimate and animate sets of words on the mean number of letters, frequency of occurrence, self-reported age of acquisition, and ratings of mental arousal, valence, dominance, and concreteness. Serra et al. (2025) later found that these words were also matched on three different measures of visual imageability.

#### 2.1.4. Procedure

A custom computer program running on desktop computers in a lab presented and recorded all materials and collected all responses. In each session, up to four participants completed the procedure on individual desktop computers at the same time. Cubicle dividers prevented participants from seeing each other’s screens during the task.

We randomly assigned participants to one of the two groups at the start of the procedure, with the restriction that we would eventually assign an even number (60) to each group. For each participant, the computer program randomly selected 60 words out of the 80 words from the Popp and Serra (2018) word list (30 animate and 30 inanimate) and put them into three lists of 20 words. Each list contained 10 animate and 10 inanimate words. None of the participants received the same word more than once. The computer program randomly ordered the words in each list for study, ignoring word type.

Before the experiment, participants completed informed consent. They then read instructions describing the basic task: that they would study and be tested on three, 20-word lists. The instructions made no mention of the animacy of the words for any of the groups. Whereas the instructions for the No Points Group made no mention of points, the instructions for the Even Points Group explained the relevant scoring system (i.e., they would earn 6 points per item correctly recalled) and urged participants to maximize how many points they earned. It read, “During study, each word will be displayed with a point value: each word will be worth 6 points if recalled correctly. Not recalling a word does not affect your score. After each test, you will be shown how many points you earned on that test, and after all three tests you will also be shown your total score for the entire task. Try to earn as many points as you can by remembering as many words as you can.”.

Participants then studied the words for the first list. Each word appeared on screen, one at a time, for 6 s with a 250 ms interstimulus interval. All words appeared in size 56, bold, Arial font with all letters being lowercase. For participants in the Even Points Group, the value of each item (6 points) appeared directly under it during study in size 48, bold, Arial font. After studying all 20 words on the first list, participants attempted to recall the words (by typing) as many of the words as they could remember. After indicating that they could not recall any more words, participants in the Even Points Group saw how many points they had earned on that list (participants in the No Points Group received no feedback). All participants then moved on to study and test over their second and third lists in the same way as their first list. After the third list, the participants in the Even Points Group saw their total score across all three lists. All participants then read a debriefing on the computer, received credit for participating, and were dismissed.

### 2.2. Results

We calculated mean free-recall performance (percent correct) for animate and inanimate items in the two groups across the three trials (Table 1). We then analyzed free-recall with a 2 (effort level: lower effort vs. higher effort) × 2 (animacy: animate items vs. inanimate items) × 3 (trial: 1 v. 2 vs. 3) mixed-effects ANOVA. Effort did not affect recall, *F*(1, 118) = 2.583, *MSE* = 1646.627, *p* = 0.111, *η_p_*^2^ = 0.021. Recall was greater for animate than inanimate words, *F*(1, 118) = 12.028, *MSE* = 180.421, *p* < 0.001, *η_p_*^2^ = 0.093. Recall differed by trial, *F*(2, 236) = 4.313, *MSE* = 276.563, *p* = 0.014, *η_p_*^2^ = 0.035. More specifically, recall was lower on the first trial than on both the second (*p* = 0.028) and third (*p* = 0.016) trial, but recall was equivalent on the second and third trial (*p* = 0.578). Animacy and trial interacted, *F*(2, 236) = 4.215, *MSE* = 201.685, *p* = 0.016, *η_p_*^2^ = 0.034, as the overall effect of animacy was significant on the second trial (*p* < 0.001), but not on the first (*p* = 0.725) or third (*p* = 0.264) trial. The interaction of trial and effort was not significant, *F*(2, 236) = 0.142, *MSE* = 276.563, *p* = 0.868, *η_p_*^2^ = 0.001, nor was the interaction of animacy, trial, and effort, *F*(2, 236) = 0.583, *MSE* = 201.685, *p* = 0.559, *η_p_*^2^ = 0.005. Most important, the interaction of animacy and effort was significant, *F*(1, 118) = 5.303, *MSE* = 180.421, *p* = 0.023, *η_p_*^2^ = 0.043. The recall of animate words was greater than that of inanimate words for the No Points Group (*p* < 0.001, *η_p_*^2^ = 0.248) but did not differ for the Even Points Group (*p* = 0.444, *η_p_*^2^ = 0.010). As well, recall of inanimate words was higher for the Even Points Group than for the No Points Group (*p* = 0.032, *η_p_*^2^ = 0.038), but recall of animate words did not differ by group (*p* = 0.405, *η_p_*^2^ = 0.006).

### 2.3. Discussion

We obtained a significant animacy advantage in Experiment 1. More critically, however, adding a value-based component for the Even Points Group led to an interaction of animacy with the presumed level of effort: the animacy advantage maintained for the No Points Group, but was eliminated for the Even Points Group. More specifically, conditions led the Even Points Group to increase their recall of inanimate words compared to the No Points Group. Recall of animate words, however, was not affected. These outcomes provide support for encoding-effort explanations of the animacy advantage.

## 3. Experiment 2

The results of Experiment 1 provided some initial support for the idea that we could use a value-directed remembering manipulation to alter the occurrence of the animacy advantage in free-recall performance. As with all of the studies in Figure 1, Experiment 1 used a between-participants manipulation of (presumed) effort. Most value-directed remembering studies, however, use within-participants manipulations of value (i.e., points or monetary rewards), allowing researchers to examine effects of higher and lower value on specific items (and note that five of the eight studies in Figure 2 used within-participants manipulation of presumed effort). We took this approach in Experiment 2, using a within-participants manipulation of point values randomly assigned to the items during encoding. Specifically, half of the animate and half of the inanimate words were worth ten points each if recalled correctly and half of the animate and half of the inanimate words were worth two points each if recalled correctly. We assumed this would result in higher and lower encoding effort, respectively. This manipulation might lead to higher recall for the ten-point than two-point items, but this outcome was not critical for the present hypothesis. More important, we predicted that animacy and value (i.e., effort) would interact such that the animacy advantage would be reduced or even eliminated for the ten-point items, much as in Experiment 1 and in the studies in Figure 1.

### 3.1. Materials and Methods

The materials and methods for Experiment 2 were largely the same as for Experiment 1, with the changes in procedure noted below.

#### 3.1.1. Design

This experiment utilized a 2 (effort level: lower effort vs. higher effort) × 2 (animacy: animate items vs. inanimate items) × 3 (trial: 1 v. 2 vs. 3) within-participants, fully factorial design. To manipulate effort, we randomly assigned half of the items to be worth 2 points if recalled correctly and half to be worth 10 points if recalled correctly.

#### 3.1.2. Participants

We attempted to use G*Power (Faul et al., 2007) to conduct an a priori power analysis to determine the target sample size for Experiment 2. This software, however, does not have a module for determining sample size for the interaction of two within-participants independent variables as in the present experiment. One simple compromise, perhaps, is to half the required sample size for Experiment 1 (i.e., from 132 to 66), as the two experiments involve basically the same design, with effort switched to a within-participants manipulation. Another option is to again use parameters from Serra et al. (2025, Study 2) as the basis for these calculations (i.e., *η_p_*^2^ = 0.093, or *f*(*U*) = 0.320) and use the next closest approximation for the present design. Under the “ANOVA: repeated measures, within factors” menu, we indicated one group and four measurements. G*Power indicated a sample size of 59 participants to achieve statistical power of 0.95 for this main effect, but note that these setting would assume one repeated-measures independent variable with four levels, not the product of a 2 × 2 factorial interaction.

We recruited 60 undergraduate students from Texas Tech University who participated for partial or extra credit in a course. As in Experiment 1, participants received credit for participating, not based on their level of performance. Mean age was 19.13 years (*SD* = 1.23). Of the participants, 47 (78.3%) identified as female and 13 (21.7%) as male. For race/ethnicity, 34 (56.7%) identified as white or Caucasian; 15 (25.0%) as Hispanic or Latino; 4 (6.7%) as multiracial; 3 (5.0%) as black or African American; 3 (5.0%) as Asian or Asian American; and 1 (1.7%) as “other”. No one omitted any demographics questions.

#### 3.1.3. Materials

Study materials were the same animate and inanimate words as in Experiment 1.

#### 3.1.4. Procedure

The procedure for Experiment 2 was essentially the same as that of the Even Points Group in Experiment 1 except that half of the animate and inanimate items on each trial were randomly assigned to be worth 2 points if recalled correctly and half were randomly assigned to be worth 10 points if recalled correctly. The point value of each item was visible during the encoding of the item. As with the Even Points Group, participants in Experiment 2 were instructed to try to maximize the number of points they earned.

### 3.2. Results

We calculated mean free-recall performance (percent correct) for animate and inanimate items for the various conditions across the three trials (Table 1). We then analyzed free-recall with a 2 (effort level: lower effort vs. higher effort) × 2 (animacy: animate items vs. inanimate items) × 3 (trial: 1 v. 2 vs. 3) repeated-measures ANOVA. Recall was greater for 10-point items than for 2-point items (i.e., effort), *F*(1, 59) = 12.685, *MSE* = 641.234, *p* < 0.001, *η_p_*^2^ = 0.177. Recall did not differ for animate and inanimate words, *F*(1, 59) = 3.260, *MSE* = 515.584, *p* = 0.076, *η_p_*^2^ = 0.052, nor did recall differ by trial, *F*(2, 118) = 2.048, *MSE* = 537.279, *p* = 0.134, *η_p_*^2^ = 0.034. Animacy and trial did not interact, *F*(2, 118) = 0.318, *MSE* = 431.573, *p* = 0.728, *η_p_*^2^ = 0.005, nor did trial and effort, *F*(2, 118) = 0.485, *MSE* = 468.465, *p* = 0.617, *η_p_*^2^ = 0.008, nor did animacy, trial, and effort, *F*(2, 118) = 1.042, *MSE* = 366.412, *p* = 0.356, *η_p_*^2^ = 0.017. Most important, however, the interaction of animacy and effort was not significant, *F*(1, 59) = 2.616, *MSE* = 430.085, *p* = 0.111, *η_p_*^2^ = 0.042.

Despite the lack of statistical significance, the effect size for the interaction of animacy and effort in Experiment 2 (*η_p_*^2^ = 0.042) was nearly identical to that of the same interaction in Experiment 1 (*η_p_*^2^ = 0.043). Given the centrality of this interaction for the present hypotheses, we conducted conceptually identical follow-up analyses as in Experiment 1 for interested readers and for empirical completeness. That said, these results should be interpreted with caution given that the critical interaction did not reach significance. The recall of animate words was greater than that of inanimate words for two-point items (*p* = 0.019, *η_p_*^2^ = 0.089) but did not differ for ten-point items (*p* = 0.808, *η_p_*^2^ = 0.001). As well, recall of inanimate words was greater for ten-point than for two-point items (*p* < 0.001, *η_p_*^2^ = 0.208), but recall of animate words did not differ by value (*p* = 0.101, *η_p_*^2^ = 0.045).

### 3.3. Discussion

**Figure 3:**
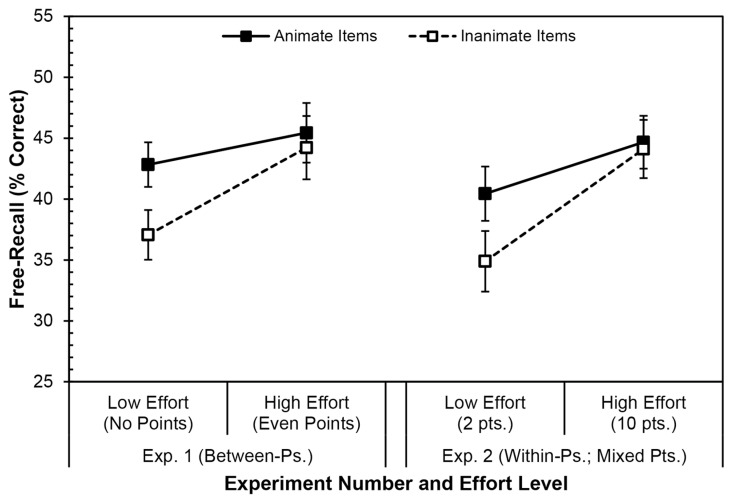
Mean free-recall performance (% correct) for animate and inanimate items, split by encoding condition, from the present experiments (collapsed on trial). Solid black lines and black boxes indicate animate items; dashed black lines and white boxes indicate inanimate items. Error bars represent one standard error of the mean.

Although switching the encoding-effort manipulation to a within-participants variable in Experiment 2 produced the same basic pattern of results as in Experiment 1 (Figure 3), the pattern of significant and non-significant results differed. First, the main effect of the within-participants value-directed remembering manipulation was significant, yielding higher recall for ten-point than two-point items. In contrast, the effect of the between-participants effort manipulation was not significant in Experiment 1. Second, the main effect of animacy and the interaction of animacy and effort were not significant, although both might be considered “marginal” (i.e., *p* = 0.076 and *p* = 0.111, respectively). In contrast, both outcomes were significant in Experiment 1. Analyzing the post hoc comparisons as in Experiment 1 yielded the same results, even without a significant interaction: higher value led to the equivalent recall of animate and inanimate items, whereas lower value led to greater recall of animate than inanimate items.

## 4. Omnibus Analysis

The inconsistent results across Experiments 1 and 2 might have resulted from differences in how the between-participants and within-participants manipulations of effort affected the participants. Another possibility is that the experiments—in particular, Experiment 2—were underpowered. As we randomly assigned participants to one of the two groups in Experiment 1 or to the single condition in Experiment 2, we can also consider the two experiments as one with three groups (i.e., the No Points and Even Points Groups, and a Mixed Points Group). Such a comparison allows us to consider if the design of the two experiments interacted with any of the other independent variables. Figure 3 presents recall from both experiments (collapsed on trial) for comparison to Figure 1 and Figure 2.

We analyzed the results with a 2 (design: between- vs. within-participants) × 2 (effort level: lower effort vs. higher effort) × 2 (animacy: animate items vs. inanimate items) linear mixed-effects regression and considered Type III Fixed Effects. The “lower effort” factor used the data from the No Points Group and the Mixed Points Group’s 2-point items; the “higher effort” factor used that from the Even Points Group and the Mixed Points Group’s 10-point items. Design did not affect recall, *F*(1, 166.293) = 0.323, *p* = 0.570, *η_p_*^2^ = 0.002. Recall was higher in higher- than lower-effort conditions, *F*(1, 295.247) = 12.862, *p* < 0.001, *η_p_*^2^ = 0.042, and was higher for animate than inanimate items, *F*(1, 300.778) = 10.068, *p* = 0.002, *η_p_*^2^ = 0.032. Importantly, the interaction between effort and animacy was significant, *F*(1, 300.778) = 5.348, *p* = 0.021, *η_p_*^2^ = 0.017. No other interactions were significant (all *p*s > 0.05).

To examine that interaction further, we conducted two separate 2 (design: between- vs. within-participants) × 2 (animacy: animate items vs. inanimate items) mixed ANOVAs (i.e., separated by effort). In the low-effort conditions, recall was higher for animate than inanimate items, *F*(1, 118) = 18.244, *p* < 0.001, *η_p_*^2^ = 0.134. Design did not affect recall, *F*(1, 118) = 0.682, *p* = 0.411, *η_p_*^2^ = 0.006, nor did animacy and design interact, *F*(1, 118) = 0.007, *p* = 0.933, *η_p_*^2^ < 0.001. In contrast, in the high-effort conditions, recall did not differ for animate and inanimate items, *F*(1, 118) = 0.424, *p* = 0.516, *η_p_*^2^ = 0.004. Design did not affect recall, *F*(1, 118) = 0.020, *p* = 0.887, *η_p_*^2^ < 0.001, nor did animacy and design interact, *F*(1, 118) = 0.060, *p* = 0.807, *η_p_*^2^ = 0.001. These results suggest that Experiment 2 was under-powered, rather than the design being the critical factor leading to the inconsistencies.

## 5. General Discussion

The present study replicated the animacy advantage in free-recall performance, such that participants were more likely to recall animate than inanimate words (cf. Bonin et al., 2015; Félix et al., 2019; Leding, 2018; Meinhardt et al., 2020; Nairne et al., 2013; Popp & Serra, 2018; Serra, 2021; VanArsdall et al., 2017). Although Experiments 1 and 2 yielded slightly different results in terms of statistical significance, the pattern of results was the same (Figure 3), and was clarified with an omnibus analysis which controlled for design. Overall, value-directed remembering led to higher recall for higher-value items and lower recall for lower-value items, indicating that these manipulations led to correspondingly different levels of encoding effort (e.g., Ariel & Castel, 2014; Castel, 2008; Murphy et al., 2024). More importantly, animacy interacted with effort: the animacy advantage was eliminated under higher-effort conditions due to increased recall of inanimate words. In contrast, a typical animacy advantage occurred under lower-effort conditions.

The present results provide further support for effortful-encoding explanations of the animacy advantage in free-recall performance (cf. Bonin et al., 2022; Leding, 2019; Meinhardt et al., 2020; Rawlinson & Kelley, 2021; Serra et al., 2025) and indicate that the animacy advantage can be reduced if participants process inanimate items more deeply during encoding. But given that some studies have not found an interaction of animacy and effort, even when effort produced a difference in recall (Figure 2), there are likely methodological or situational factors that moderate this relationship that can be explored in future research to better understand the causes and boundaries of this effect.

### 5.1. Resolving Inconsistent Findings Across Studies

Considering the present results along with those of past studies that have provided evidence for or against the effortful-encoding explanation for the animacy advantage, it might at first seem difficult to decide whether this explanation is supported or not. We argue that considering additional factors might help to explain why the expected interaction of animacy and effort occurs in some studies but not others. We consider several such factors here, although this is not likely an exhaustive list.

Our omnibus analysis of the present experiments suggests that whether we manipulated effort between- or within-participants did not alter the results, but nevertheless this design choice could still contribute to the inconsistencies across previous studies. Considering the studies in Figure 1 and Figure 2, all six studies in Figure 1 involved a between-participants manipulation of presumed effort and all six found an interaction between animacy and that variable. In contrast, the eight studies in Figure 2 did not find an interaction between animacy and effort. Of those, three involved a between-participants manipulation of presumed effort (Figure 2a,b,h) and five involved a within-participants manipulation (Figure 2c–g). The interaction of animacy and effort might therefore be more likely to occur under mixed designs with effort as a between-participants manipulation. When effort is manipulated between participants, participants under higher-effort conditions might be more likely to apply consistent encoding effort across all items, regardless of the animacy of those items. But when effort is manipulated within participants, participants might apply greater encoding effort across all items assigned to the higher-effort condition and consistently reduced encoding effort across all items assigned to the lower-effort condition (regardless of their animacy in both cases). This explanation lacks a clear mechanism, but it describes the patterns in Figure 2c–g well.

Inconsistent manipulations of effort across studies might have led to inconsistent interactions with animacy: the levels of the manipulation might not have differed enough to produce an interaction. For example, consider that for Leding (2018, Study 1, Figure 2a), a pleasantness rating was the “more effortful” encoding condition (compared to looking for the letter e in the target words), but for Blunt and VanArsdall (2021, Study 1, Figure 1d), a pleasantness rating was the “less effortful” encoding condition (compared to the method of loci). The pleasantness rating produced an animacy advantage in both studies, and the level of recall was about the same in both. Only Blunt and VanArsdall (2021, Study 1), however, produced a significant interaction of animacy with effort. Rating the pleasantness of the words and looking for the letter e in the words might not have involved a large enough difference in encoding effort to produce the relevant interaction. Such findings suggest that there might need to be a larger difference in the levels of processing for the interaction to obtain. The “lower effort” condition might indeed need to involve a very low level of effort, and the “higher effort” condition a very high level of effort.

Similarly, most of the studies in Figure 1 and Figure 2 involved incidental encoding conditions and surprise memory tests. The effortful-encoding explanation does not require that the encoding is purposeful; most of the earlier studies supporting this account assumed that the effort was a spurious byproduct of animate concepts (i.e., Meinhardt et al., 2020; Rawlinson & Kelley, 2021). Although the animacy advantage occurs under both incidental and intentional learning conditions, it tends to be larger under incidental conditions. Consider that the critical interaction of animacy and effort occurred for Serra et al. (2025, Study 2, Figure 1f), which involved incidental learning, but not for Serra et al. (2025, Study 1, Figure 2h), which involved intentional learning. Under intentional learning conditions, a smaller magnitude of animacy advantage and overall greater effort devoted to encoding items might make the interaction of animacy and effort less likely to obtain. That said, some studies have involved intentional learning and found the interaction, such as Blunt and VanArsdall (2021, Study 1, Figure 1d) and the present Experiment 1. In contrast to their first study, Blunt and VanArsdall (2021, Study 2, Figure 2d) also used intentional learning but failed to obtain the interaction. The pattern of that study (Figure 2d) is nearly identical to that of Serra et al. (2025, Study 1, Figure 2h) in that there is little difference in the level of recall for the conditions that presumably involved higher and lower levels of processing, and an overall small animacy advantage. Nevertheless, the interaction might simply be more likely to obtain with incidental learning. As well, although it is not always the case, in three of the studies in Figure 1c,e,f, the interaction seems to reflect not only an increase in the recall of inanimate items, but also an apparent (albeit not significant) decrease in the recall of animate items in the (presumably) more effortful condition compared to the less effortful condition. This outcome can only be explained by effortful-encoding accounts: when devoting extra attention and effort to the processing of the inanimate items, the processing of the animate items might be consequently reduced. Such outcomes might be more likely with intentional learning conditions; two of those three studies (Figure 1c,f) involved intentional learning.

Another possibility is that the level of recall performance makes it more or less possible for the animacy advantage—or the interaction of animacy with effort—to obtain. Studies of the animacy advantage use a fixed number of animate and inanimate items. As the overall level of recall increases, the recall of both animate and inanimate items also increases (cf. relevant analyses in Serra et al., 2025). As there are a finite number of items to recall, the animacy advantage will often be largest when the overall level of recall is low and smallest when the overall level of recall is high (Serra et al., 2025). The level of recall by condition might therefore limit or increase the chance that an interaction of animacy and effort can occur in a given study. A related possibility involves the difficulty of the items or the task themselves. Not only might item or task difficulty affect the level of recall performance, but they might also make some encoding strategies more or less effective. If the items are very difficult to recall or the task demands limit recall (e.g., too many items to recall or a too-fast presentation rate), then participants might not be able to employ more effortful encoding strategies, and the interaction might not obtain.

Some studies of the animacy advantage have considered factors of the task or of the participants that might affect how participants interact with the items in a top-down way. For example, although DeYoung and Serra (2021) provided strong evidence that people’s metacognitive beliefs about animacy and memory do not contribute to the occurrence of the effect under computer-paced conditions, Serra and DeYoung (2023a) found that such beliefs can alter the effect under self-paced encoding conditions. Specifically, participants who believed that inanimate items would be easier to recall than animate items studied all items for longer than did other participants, allocated equal study time to the animate and inanimate items, and later showed equivalent recall for animate and inanimate items. In other words, their belief that inanimate items would be more memorable led them to devote more time (and perhaps effort) to encoding all the items, eliminating the animacy advantage for those participants. In an unpublished study, Shull et al. (2022) instructed some participants to focus on encoding animate over inanimate items and some to focus on encoding inanimate over animate items; a control group received no instructions. The control group recalled more animate than inanimate words, *t*(39) = 2.295, *p* = 0.027, *Cohen’s d* = 0.36, but the effect was much larger for the group told to focus on the animate items, *t*(39) = 7.448, *p* < 0.001, *Cohen’s d* = 1.18. In contrast, the group told to focus on the inanimate items recalled more inanimate than animate words, *t*(39) = 2.796, *p* = 0.004, *Cohen’s d* = 0.44, although the size of this effect fell between those of the other two groups, suggesting that it is somewhat easier to devote greater attention or effort to encoding animate than inanimate items. In Komar et al.’s (2024) Study 4, participants rated the relevance of animate and inanimate words for achieving either three survival-related goals or one survival-related goal. Although they found an interaction of animacy with encoding effort, follow-up analyses indicated that the animacy advantage was reduced for words that were rated as more relevant compared to those rated as less relevant. Adding relevance as a factor to their analyses eliminated the interaction of animacy with encoding effort. Although disparate in nature, these outcomes are all consistent with encoding-effort accounts of the animacy advantage and suggest that it is possible to show additional evidence for encoding-effort explanations under more contrived situations. As well, they indicate that there might be undetected or “embedded” interactions within many studies of the animacy advantage that can alter the occurrence of the effect but that might not be discernible unless those factors are explored explicitly.

The fact that specific conditions of experiments moderate the contribution of effortful encoding to the animacy advantage does not rule out this explanation, but it suggests that the effect is not likely caused by one single mechanism (DeYoung & Serra, 2021; Serra et al., 2025). It might even arise for different reasons under different conditions or for different participants (e.g., Serra & DeYoung, 2023a; but see Serra et al., 2025). Further research is still needed to fully understand all the mechanisms that contribute to the effect or the conditions under which some factors play a larger or smaller role.

### 5.2. Implications for Adaptive-Memory Accounts

Adaptive memory theories suggest that human memory systems were tuned by evolutionary pressures to prioritize biologically relevant information such as living things because remembering such information conferred survival advantages (e.g., Nairne et al., 2013; Nairne & Pandeirada, 2016; Serra et al., 2024). Under this framework, the animacy advantage is assumed to be a built-in bias: animate stimuli automatically receive privileged encoding or retention due to their relevance for threat detection, predator–prey dynamics, social interaction, or ecological utility (e.g., Nairne et al., 2013; VanArsdall et al., 2015, 2017; Leding, 2020). The present findings, however, indicate that the animacy advantage can be altered by task demands: when participants are induced to invest extra encoding effort to inanimate items via mental-imagery instructions or value-directed remembering, the effect can be attenuated or even eliminated. Such findings do not rule out adaptive accounts of the effect but instead help to elucidate the specific mechanisms that might cause it to occur in modern humans.

## Figures and Tables

**Table 1:** Mean recall performance (percent correct) by item type, condition, and trial ^1^.

	Trial 1	Trial 2	Trial 3
Condition and Measure	*M* (*SD*)	*M* (*SD*)	*M* (*SD*)
Experiment 1 (Effort Manipulated Between-Ps)			
No Points Group (Lower Effort)			
Animate Recall	38.83 (17.86)	44.83 (20.46)	44.83 (16.72)
Inanimate Recall	36.17 (19.32)	36.00 (20.27)	39.00 (21.68)
Animacy Advantage	+2.66	+8.83	+5.83
Even Points Group (Higher Effort)			
Animate Recall	41.50 (23.64)	49.50 (23.17)	45.17 (21.75)
Inanimate Recall	42.83 (22.48)	42.83 (24.98)	47.00 (24.45)
Animacy Advantage	−1.33	+6.67	−1.83
Experiment 2 (Effort Manipulated Within-Ps)			
2-Point Items (Lower Effort)			
Animate Recall	38.67 (25.21)	41.67 (23.08)	41.00 (25.36)
Inanimate Recall	30.00 (22.25)	34.67 (27.28)	40.00 (26.55)
Animacy Advantage	+8.67	+7.00	+1.00
10-Point Items (Higher Effort)			
Animate Recall	43.00 (27.27)	44.67 (23.10)	46.33 (22.85)
Inanimate Recall	43.33 (24.19)	44.33 (26.32)	44.67 (25.87)
Animacy Advantage	−0.33	+0.34	+1.66

^1^ Recall values are the mean percentage of items of a given type correctly recalled on each study-test trial; values in parentheses are the standard deviation of that mean. “Animacy Advantage” values are the signed difference in the animate and inanimate recall.

## Data Availability

All materials, data, code, and protocols from the experiment are available on OSF at https://osf.io/zthx2/overview?view_only=948f998168fd4604a48a8f90f4f549b6 (accessed on 29 October 2025).

## Abbreviations

The following abbreviations are used in this manuscript:

EEG	Electroencephalography
ERP	Event-Related Potential
